# Quality of life, coping strategies and support needs of women seeking Traditional Chinese Medicine for infertility and viable pregnancy in Australia: a mixed methods approach

**DOI:** 10.1186/1472-6874-13-17

**Published:** 2013-04-09

**Authors:** Karin Ried, Ann Alfred

**Affiliations:** 1 Discipline of General Practice, The University of Adelaide, Adelaide, South Australia, 5005,, Australia; 2 National Institute of Integrative Medicine, Melbourne,, Victoria, 3123, Australia

**Keywords:** Infertility, Quality of life, Coping, Social support, Fertility awareness, Traditional Chinese medicine

## Abstract

**Background:**

Infertility affects about 15% of couples in Western-societies with most progressing to fertility clinics for treatment. Despite being common, infertility is often experienced as a lonely road for affected couples. In this paper we expand on our previously published findings of women’s experiences with infertility or difficulty of viable pregnancy who had sought Traditional Chinese Medicine (TCM) therapy in Australia, and focus on women’s quality of life, coping strategies, and support needs.

**Methods:**

We applied mixed methods using the Tuebingen Quality of Life and the COPE questionnaires and in-depth interviews with 25 women with primary or secondary infertility, recurrent miscarriages or unexplained stillbirth, and who had consulted a TCM practitioner. We used a thematic approach to analyse the interviews, and descriptive statistics to evaluate questionnaire responses.

**Results:**

Women reported through both questionnaires and interviews compromised quality of life due to the high level of distress, guilt, grief, and frustration caused by infertility. However, our women represented a highly motivated sample, actively seeking alternative support. While the TCM approach to infertility management increased women’s sense of personal agency and control through education and continuity of care, the need for greater understanding and support on a societal level remains.

**Conclusions:**

In infertility, ongoing emotional and instrumental support is pivotal to the wellbeing and quality of life of the affected. Traditional Chinese Medicine addresses some support needs in infertility not routinely available in the Western model of care. More peer-led and professional-led support groups are greatly needed for women experiencing infertility to help break isolation and raise awareness of integrative approaches to fertility management.

## Background

Infertility, defined clinically as the failure to conceive after one year of unprotected intercourse or demographically as the inability to achieve a live birth [1-3], affects a large number of couples in Western societies. In Australia, 15% or 3 million couples currently deal with impaired fertility, which might be related to male or female factors or remains ‘unexplained’ in 28% of cases [4,5].

Most couples in Australia seeking treatment for infertility are referred by their General Practitioner to assisted reproductive technology (ART), including in vitro fertilization (IVF). However, of 70,500 ART cycles performed in Australia and New Zealand in 2009, only 17.2% led to live births [4]. Moreover, ART treatment is costly for both government and individuals; in 2009, the Australian Government spent approximately A$250 million for ART services [4,6,7], and individual couples’ out-of-pocket expenses were about A$3,000 for one IVF cycle costing A$7,000-8,000 [8].

Despite infertility occurring relatively often in the population, it is experienced as a lonely road for individual couples [9-11]. In addition to isolation, infertility challenges women’s sense of identity, expectations of their life trajectory and their perceived value in society [9,11]. This can lead to feelings of failure, guilt and shame [11-13]. Women’s sense of powerlessness can be exacerbated by the Western medical approach, through fragmented care, technical interventions and invasive procedures, and a lack of individualised continuous support [14].

Holistic approaches to infertility management, such as Traditional Chinese Medicine (TCM) might address some of the needs of women experiencing infertility, not met in the Western Medical approach [15-17]. Traditional Chinese Medicine treatment encompasses herbal medicines, acupuncture and lifestyle counselling based on the individual’s underlying TCM pattern diagnosis using tools such as pulse, tongue, general physical and emotional wellbeing, and menstrual history [16,17]. In TCM, different conditions such as idiopathic infertility, polycystic ovaries, recurrent miscarriage or unexplained stillbirth, may have similar underlying TCM pattern (e.g. Kidney Yin Deficiency Heat), and treatment would therefore be approached with similar therapies [16,17].

Recent meta-analyses of randomised controlled trials of TCM herbal therapy for female infertility revealed a 2 to 3.5-fold higher likelihood of pregnancy within a 4-month treatment period compared with Western Medical drug therapy [18,19]. In addition, a meta-analysis of cohort studies involving more than 600 women suggested a mean clinical pregnancy rate of 50% using Chinese herbal medicine [18]. While complementary and alternative medicines are increasingly used for infertility in western countries, herbal medicines including TCM herbal treatment, are being used only by a small proportion of affected women, e.g. 5% of those surveyed at an infertility clinic in South Australia, 10% in the UK, 18% in the USA [20,23]. In contrast to the better known and increasingly established use of acupuncture as an adjunct treatment to IVF [24], the level of communication about and awareness of holistic TCM therapy for fertility management is low, with most women hearing about TCM as an alternative and complementary treatment option only through word of mouth [14,25].

Our study is among the first to examine women’s experience of holistic TCM therapy for infertility and viable pregnancy. In this paper, we expand on our previously reported findings describing women’s experience of infertility and TCM treatment and outcomes, and explore specifically the quality of life, coping strategies and emotional and instrumental support needs of women with infertility or difficulty of viable pregnancy seeking TCM therapy.

## Methods

### Participants

A purposive sample of 25 women with infertility or difficulty of viable pregnancy participated. Women had consulted a TCM practitioner for 1–6 months at the time of the interview. We included women aged 20–45 years with primary (nulliparous) or secondary infertility (primi- or biparous), recurrent miscarriages (≥ 3 gravida) or unexplained stillbirth (>20 weeks gestation), in accordance with the broader definition of infertility or difficulty of achieving a life birth [3]. Women may have received treatment in fertility clinics. We excluded women whose partner had been diagnosed with male factor infertility.

We recruited Australia-wide between November 2008 and February 2010, through TCM practitioners, Internet forums, and newspaper advertisements. Recruitment ceased when thematic saturation was reached [26]. Twenty-six women expressed interest in participating in the study by phone or email. Study information, demographic data forms, consent forms and reply paid envelopes were mailed before the interviews to screen for eligibility. One women was not eligible due to multiparity >3. Twenty-five women were eligible and provided written informed consent before participation. The study protocol was approved by the University of Adelaide Human Research Ethics Committee.

### Study design and setting

We applied a mixed methods approach, using questionnaires and semi-structured in-depth interviews, and used thematic analysis to explore the quality of life, coping strategies and support mechanisms of women with infertility seeking TCM therapy. We also explored women’s experiences with TCM therapy itself which we have described in detail elsewhere [14].

### Interviews

The hour-long interviews were conducted by a qualified, experienced female counsellor/researcher (AA) face-to-face at the University of Adelaide for women living in South Australia and by telephone for interstate participants. An interview schedule with the main topics on quality of life, coping strategies, support mechanism, and experience of menstrual health, infertility, treatment approaches, guided the interviews, but participants were encouraged to elaborate freely.

### Questionnaires

In addition, all participants were given two questionnaires after the interview, which they filled out in their own time and returned to the study centre in a reply paid envelope. One questionnaire assessed the quality of life and the second questionnaire assessed women’s coping strategies at the time of TCM therapy. Women reflecting on their experience were given the questionnaires in the past tense, and women undergoing TCM therapy at the time of the interview were given the questionnaires in the present tense.

### Quality of life questionnaire

Quality of life was assessed using the English short version of the ‘Tuebingen Quality of Life Questionnaire’ (TLMK) adapted for females [27,28]. Our questionnaire consisted of 29 items which explored multiple areas, including desire for a child and associated stress, marital relationship, gender identity, and psychological well-being on a 5-point Likert scale ranging from’1=strongly disagree’ to ‘5=strongly agree’ (Additional file 1).

### Coping questionnaire

To assess the coping strategies of women with infertility, we used the short form of the validated multidimensional coping inventory ‘COPE’ [29]. The 53-item questionnaire assessed 14 ways of coping: positive reinterpretation and growth, active coping, planning, mental disengagement or distraction, venting of emotions, acceptance, denial, behavioural disengagement or giving up, restraint, suppression of other activities, seeking instrumental social support/advice, seeking emotional support, religious coping, and substance abuse. Each way of coping was assessed by four non-consecutive statements on a 4-point Likert-scale ranging from 1= ‘I usually don’t do this at all’ to 4= ‘I usually do this a lot’.

### Analysis

The interviews were audiotape, transcribed and data coded independently by the two authors (KR, AA) and two students (AN, EL) using a thematic approach. Transcription was reviewed for accuracy against the audiotapes. Initial coding was guided by the interview schedule and augmented by women’s stories. Coding was refined by repeated reading of transcripts and organised into themes using NVivo 8. Key themes were identified independently and any disagreements resolved in discussion between the authors.

Questionnaire responses were validated with the interviews, and analysed using the SPSS Statistics program version 18.0.2 [30].

For the *Quality of Life questionnaire*, we calculated the mean score for all participants and subgroups of women with primary infertility, secondary infertility, or stillbirth for each of the items. Proportions of combined positive responses (strongly agree + agree) were calculated for all participants and for the largest subgroup of women with primary infertility.

For the *COPE questionnaire* we calculated the mean score (SD) for each of the 14 ways of coping subscales with one subscale consisting of up to 4 statements [29]. Scores of our group of women were normally distributed and comparable between the subgroups.

## Results

### Characteristics of participants

Two-thirds of women in our study were recruited through TCM practitioners, the remainder through internet forums, newspaper advertisements, and by other participants Australia-wide. The majority of women (mean age 37.3 years) had postgraduate qualifications (n=19, 76%), and were born in Australia (n=20, 80%) (Table 1). Most women in our study had problems with conception (n=19, 76%), and one-fifth had not been able to carry a pregnancy to term (n=6, 24%). Four women had secondary infertility. Two-thirds (n=17, 68%) of women had attended a fertility clinic and then proceeded to TCM, of those 14 (56%) had been unsuccessful with IVF and sought other treatment options. Others (n=8, 32%) chose to try TCM first.

**Table 1:** Characteristics of participants

**Characteristics**		**N**	**%**
**Recruitment**	TCM practitioners (n=3)	16	64
	Internet forums (n=2)	3	12
	Newspaper advertisements	2	8
	Other participants	4	16

**Mean age**	37.3 years (SD 3.6)	25	100
**Education**	University degree	14	56
	Vocational	5	20
	Secondary school	6	24

**Ethnicity**	Caucasian	23	92
	Latin American	2	8
**Place of birth**	Australia	20	80
	Overseas English-speaking	3	12
	Overseas non English-speaking	2	8

**Place of residence**	South Australia	22	88
	Other state in Australia	3	12
**Infertility status**	Primary infertility	18	72
	Secondary infertility	4	16
	Stillbirth	3	12

**Infertility history**	**Problems with conception:**	19	76
	Unexplained infertility	11	44
	PCOS	4	16
	Endometriosis	3	12
	Poor egg quality	1	4
	
	**Problems retaining viable pregnancy:**	6	24
	Recurrent miscarriage (range n= 3–5)	3	12
	Stillbirth	3	12

**Mean (range) time trying to conceive**	4.5 (1–10) years	25	100
**Conventional/WM treatment**	Attended fertility clinic	17	68
	Number of IVF cycles (mean 2.5, range 0–15)	14	56

*SD*, standard deviation, *PCOS*, polycystic ovarian syndrome, *WM*, Western medicine, *IVF*, in vitro fertilisation.

### Quality of life

Three-quarters (76%) of all women reported a high level of distress related to not having been able to have a child, and half of all women felt guilty or hurt when others made remarks about their childlessness (Table 2). The majority of women in our study experienced primary infertility (n=18). Of these women, 83% felt distressed, half reported feeling ‘like a failure’ and being under pressure around ovulation, and more than a third considered infertility a personal shortcoming, reported being upset when they saw a baby carriage, or recognized that life revolved around trying to have a child.

**Table 2:** Quality of life

**Questions**^**1**^	**All women**	**Primary/Secondary infertility**	**All women**	**Subgroup: primary infertility**	**Subgroup: secondary infertility**	**Subgroup: still birth**
**Number of participants**	**n=25**	**n=18/4**	**n=25**	**n=18**	**n=4**	**n=3**
	**%agree**^**2**^	**%agree**^**2**^	**mean (SD)**^**3**^	**mean (SD)**^**3**^	**mean (SD)**^**3**^	**mean (SD)**^**3**^
Not having been able to mother a child is distressing to me.	**76**	**83/50**	**3.8 (1.1)**	**4.0 (1.1)**	**3.0 (1.4)**	**3.7(0.6)**
I feel guilty for having let my partner down.	**48**	**50/50**	**3.0 (1.4)**	**2.9 (1.5)**	**2.8 (1.5)**	**3.3(0.6)**
I feel hurt when others make remarks about our childlessness.	**48**	**45/50**	**3.1 (1.1)**	**3.2 (1.1)**	**2.8 (1.5)**	**3.3 (1.2)**
I feel like a failure because of our problems conceiving.	**48**	**50/25**	**2.8 (1.5)**	**2.9 (1.5)**	**2.0 (1.4)**	**3.7 (0.6)**
My partner and I have less sex when I am not ovulating.	**44**	**50/25**	**3.0 (1.4)**	**3.2 (1.4)**	**2.5 (1.3)**	**2.3 (1.5)**
I feel under pressure when I am ovulating.	**40**	**45/25**	**3.1 (1.2)**	**3.2 (1.4)**	**2.8 (1.7)**	**3.0 (1.0)**
I feel down.	**48**	**33/75**	**3.0 (1.1)**	**2.9 (1.1)**	**3.3 (1.5)**	**3.3 (1.2)**
My life revolves around trying to have children.	**36**	**39/67**	**2.8 (1.2)**	**3.0 (1.3)**	**1.8 (0.5)**	**3.3 (1.2)**
I feel less satisfied after sex than I used to before we were trying to conceive.	**40**	**39/50**	**2.6 (1.3)**	**2.7 (1.3)**	**2.8 (1.5)**	**2.3 (1.5)**
I consider infertility a personal shortcoming.	**36**	**39/-**	**2.8 (1.4)**	**2.8 (1.4)**	**1.8 (1.0)**	**4.0 (1.0)**
I feel upset when I see a baby pusher/stroller.	**36**	**33/25**	**2.7 (1.3)**	**2.6 (1.4)**	**2.5 (1.7)**	**3.7 (0.6)**
Planning of our future has been hindered by our difficulties conceiving.	**28**	**33/-**	**2.6 (1.4)**	**2.8 (1.5)**	**1.5 (0.6)**	**2.7 (1.2)**

*SD*, standard deviation.

^1^Table summarises only those questions of the 29-item questionnaire to which more than 30% of the primary infertility subgroup responded with agree or strongly agree.

^2^Combined responses ‘agree’ or ‘strongly agree’.

^3^Each item of the quality of life questionnaire was assessed on a 5-point Likert scale ranging from 1 = ‘strongly agree’ to 5= ‘strongly disagree’. Greater mean values are associated with lower quality of life.

Mean scores of women with primary infertility in our study (n=18) tended to be higher compared to mean scores of all women in our study including women with secondary infertility (n=4) and lower than for women who experienced stillbirth (n=3), with higher levels being associated with lower quality of life (Table 2).

Quality of life was also explored in the interviews. Women expressed a variety of negative emotions, including guilt, disappointment at oneself, frustration, fear of disappointment, self-blame, grief, devastation, anxiety, sadness and depression.

I have a lot of guilt associated with not being able to get pregnant, guilt for my partner, guilt for my parents. (ID 2, primary infertility, aged 36 years)

I’m frustrated my body won’t do what I want it to do. (ID 15, secondary infertility, aged 38 years)

I blamed myself more than anything. When the pregnancy didn’t work, it was my fault (ID 12, stillbirth, aged 38 years)

I feel like half a woman. (ID 2, primary infertility, aged 36 years)

These negative emotions had implications beyond personal identity and the relationship with the partner and often would influence social life and work relationships.

I’ve avoided little kids’ birthdays. I’ve cut back on socialising. Small talk is hard, because ‘what have you been doing?’ Well, trying for a baby is all I’ve been doing. That’s consumed my life for the last year, so it’s hard to chat about meaningless things. (ID 24, primary infertility, aged 37 years)

Some participants expressed their frustration with friends and society, either as envy or as anger at others’ lack of understanding.

I’ve seen women, they’re so much bigger than me and I think how come they’ve fallen pregnant and I can’t. (ID 16, primary infertility, aged 38 years)

It’s not fair, that some drunk or smoking women have babies. I don’t drink, I don’t smoke. (ID 26, primary infertility, aged 35 years)

Having friends say ‘just relax’ when they’ve had no idea because they’ve not been through it themselves. (ID 3, primary infertility, aged 33 years)

About half of the women highlighted their need not to disclose their infertility to friends or family, for some disclosure about fertility problems was a double-edged sword, potentially creating extra pressure and expectations. Some women felt being pushed from friends or family towards IVF as the only treatment solution to their problem but did not necessarily agree with this.

I felt I was this infertility thing walking around and people could see, people could tell. (ID 2, primary infertility, aged 36 years)

I also don’t want people looking down, Oh is she pregnant?, just had a little bit of a bloated stomach, had an extra piece of cheesecake, or thinking Oh she’s moody. She must be on the hormones. (ID 22, primary infertility, aged 36 years)

I don’t want the pressure of mum knowing. If she knew that we were trying she’d be asking all the time, but I want to do this with my husband and be on my own timeline, not her timeline. (ID 25, primary infertility, aged 33 years)

It (IVF) is just the accepted form of treatment. People think if you don’t have success falling pregnant, you will automatically go on to IVF. My husband is not particularly keen on IVF…he worries about the effect of those artificial hormones on my body, what affect it that going to have in 10 years. .. In the end it’s my choice, it’s not something that I’m obliged to do. (ID 13, primary infertility, aged 37 years)

Two-thirds of the women in our study had attended a fertility clinic before seeking TCM, with most stating that dealing with treatment in fertility clinics was emotionally challenging. Women expressed feeling uncomfortable or fearful, and viewed the IVF process as intrusive, time consuming or left alone with negative news.

I hate going in there. To me the place is full of misery. (ID 2, primary infertility, aged 36 years)

It’s time consuming, going in there, having a blood test, taking hours off (work) to have a transfer,… (ID 22, primary infertility, aged 36 years)

I’d been told over the phone that it was unsuccessful. I finish the conversation and I start to cry in the middle of the road. I’m on my own. (ID 14, primary infertility, aged 41 years)

Women highlighted that infertility comes with costs, financially, emotionally, socially and with regard to life plans.

The financial side puts a huge strain – and everything is on hold. My husband said recently: “Babe, I want my life back.” (ID 8, primary infertility, aged 39 years)

Trying to conceive, it’s a roller coaster of emotions, you get positive at the start of your cycle and then your period comes and you hit a wall. It takes over your life completely. (ID 9, stillbirth, aged 32 years)

Life feels like it is put on hold whilst undergoing treatment as it may take a long time and you don’t want to make decisions such as holidays or leaving town or purchasing furniture for a room that could be used as a baby room. (ID 19, primary infertility, aged 37 years)

### Coping strategies

Unsurprisingly, in our group of women who had actively sought TCM therapy as an option for infertility treatment, ‘active coping’ and ‘planning’ were the most frequently reported coping styles, followed by ‘seeking advice’, ‘personal growth’ and ‘seeking emotional support’. All women indicated that they were not ready to ‘accept’ infertility, nor had they withdrawn from other activities in life (Table 3).

**Table 3:** Coping strategies

**Ways of coping subscales**	**All women (n=25)**	
	**Interpretation: “I usually do this …”**	**Mean (SD)**
Active coping = doing something about it	Medium – a lot	3.31 (0.56)
Planning	Medium – a lot	3.16 (0.67)
Seeking advice = instrumental social support	Little – medium	2.71 (0.96)
Positive reinterpretation and growth	Little - medium	2.66 (0.94)
Seeking empathy/discussing = emotional social support	Little – medium	2.63 (1.05)
Acceptance	Little	2.19 (0.95)
Venting emotions	Little	2.06 (0.98)
Restraint = Restraining oneself of doing other things	Little	2.00 (0.78)
Religious coping	Little	2.00 (1.16)
Suppress doing other things	Little	1.97 (0.63)
Mental disengagement/distraction	Little	1.91 (0.61)
Giving up = behavioural disengagement	Not at all – little	1.33 (0.64)
Denial	Not at all – little	1.33 (0.64)
Substance abuse	Not at all (all participants)	1.00 (0)

*ART*, Assisted reproductive technologies.

Each way of coping subscale was assessed by four non-consecutive statements on a 4-point Likert scale ranging from 1 = ‘I usually don’t do this at all’ to 4 = ‘I usually do this a lot’. The statements are listed in Carver 1998.

Women engaged in activities which helped them cope with their situation by providing emotional support and reducing stress, including yoga or meditation (n=9, 36%), gym sessions or taking long walks (n=7, 28%), self-hypnosis, doing puzzles, shiatsu, or kinesiology. A third of the women had sought information about infertility treatment online, engaged in internet forums, and read recommended books, which also helped break their isolation and provided emotional support. Some women found the media (TV or radio interviews with a fertility expert) helpful.

Disheartened by difficulty achieving a viable pregnancy, a third of the women had sought information on adoption, donor eggs or surrogacy (n=9, 36%).

### Support mechanisms

Most but not all women felt supported emotionally and practically by their husband, family and others (Table 4).

**Table 4:** Support mechanisms

**Support mechanisms**	**All women, n= 25**	
**a) Emotional support**	**Supportive**	**Not supportive/non-disclosure**
Husband	22 (88%)	3 (12%)
Family	18 (72%)	5 (20%)
Mother	7	3
Sister	4	-
Father	-	1
Mother-in-law	-	1
Close friends	22 (88%)	5 (12%)
Work colleagues	6 (24%)	2 (8%)
Pets	1 (4%)	-
Counsellor at fertility clinic	1 (4%)	4 (16%)
Peer-led local support group	3 (12%) infertility support group	19 (76%) did not know any groups
Online discussion forums	5 (20%)	20 (80%) did not seek online forums
Professional-led support & peer-led support program	3 (12%) for stillbirth e.g. SIDS & Kids, Teddy Love Club, SANDS	22 (88%) did not know any local or national groups for infertility & early pregnancy loss
**b) Instrumental support**		
TCM practitioner/naturopath (fertility awareness education, lifestyle advice)	25 (100%) incl. 17 who also attended Western fertility clinics beforehand	
Books on natural fertility	5 (20%)	
Online forums (unsolicited)	5 (20%)	
GP referrals to TCM practitioner	1 (4%)	
	1 (4%) acupuncture as adjunct to IVF	

*GP*, general practitioner, *TCM*, Traditional Chinese medicine, *IVF*, in vitro fertilization.

Some women expressed the lack of support by their partner or family:

My husband said things like, ‘We don’t have to have kids. We have a fantastic flat and we’ll have plenty of money.’ I don’t want a lot of money and a great flat, I want a baby. It wasn’t important to me all that stuff. I thought we’re quite a long way apart on this. It actually made me quite annoyed. (ID 1, primary infertility, aged 41 years)

I was conscious of the fact that it would have been their first grandchild … so we didn’t discuss my problems for a long time. Since then, my brother and his wife were pregnant and had a baby and I felt more able to bring it up knowing there was already a first grandchild on the scene. (ID 7, primary infertility, aged 31 years)

While some women appreciated that counselling support was available at the treating fertility clinic, they described this primarily as grief counselling but had hoped for advice on alternative paths they could explore.

I walked into the counsellor’s office and she had nothing, no other alternatives to offer me. I was expecting for her to say, ‘I’ve got a list of ten naturopaths you can go and see or you can go and try yoga and meditation, you can go and do this or that.’ She was more like a grief counsellor like helping you get over the fact that it was never going to happen. (ID 21, primary infertility, aged 44 years)

Sometimes I wonder whether it would have helped to have talked to someone who had actually gone through it rather than a counsellor. (ID 19, primary infertility, aged 37 years)

### Professional-led support by TCM practitioners

A consultation with a Traditional Chinese medicine practitioner entailed enquiry of women’s general health, menstrual history, lifestyle characteristics such as diet, and examination of pulse and tongue. Menstrual health assessed by the quality of the menstrual cycle was particularly informative for the assessment of fertility status, and included basal body temperature (BBT) pattern, the quality of menstruation manifested by colour, flow, length and frequency, as well as mucus quality and experience of any premenstrual symptoms such as pain or breast distension. Menstrual cycle characteristics are associated with TCM pattern which can be treated accordingly [16,18].

In contrast to their experience with fertility clinics, women felt emotionally supported by the holistic approach of their TCM practitioner in all stages, from preconception to and throughout pregnancy. The holistic approach included non-invasive physiological evaluation, diet and lifestyle assessment and advice, education about the menstrual cycle and was supported by continuity of care. As a consequence, women felt respected and empowered.

The TCM practitioner looks more at your whole body. She is not overly interested in one specific area, whereas at the gynaecologist it’s very specific. (ID 15, secondary infertility, aged 38 years)

The TCM practitioner gave me some things to do, gave me herbs and recommended what foods to eat.... I feel it puts the power back in my hands, that I can do something constructive. (ID 10, secondary infertility, aged 36 years)

With TCM you’ve got that constant history. She wants to know how I have been feeling, what’s going on. You’re seeing one person for everything. (ID 17, secondary infertility, aged 41 years)

We talked about why I want to get pregnant, why is it so important to me, what am I doing in my life now, what can I clear out. (ID 21, primary infertility, aged 44 years)

Women regained hope for a solution to their infertility, particularly those women ‘diagnosed’ with unexplained infertility in the Western model of care (n=11, 44%).

The doctors seemed to have washed their hands off me because there was nothing visibly wrong with me. I don’t believe in unexplained, there‘s got to be a reason (ID 25, primary infertility, aged 33 years)

All women in the study received Chinese herbal remedies, often in combination with acupuncture, as well as lifestyle and dietary advice according to TCM diagnostic principles. Women were advised that regulation of the menstrual cycle could indicate enhanced quality of the reproductive environment, including the oocytes and endometrium, and therefore increase the likelihood of conception and implantation. In addition, women learned that diet can play a pivotal role in influencing fertility, in accordance with the literature [16,31].

All women reported menstrual changes after 1–3 months of TCM therapy, including the regulation of temperature pattern and cycle length or changes to blood and mucus quality, indicative of improved fertility and chance of viable pregnancy [14]. Within one year after the interviews, 13 (52%) women had given birth to healthy babies, six of those after TCM treatment alone, and seven with IVF after TCM therapy [14]. Follow-up of participants was ceased after one year.

### Peer-led support groups

Outside the support by the TCM practitioner, our group of women unanimously expressed feeling understood only by those who had had similar experiences with infertility. Therefore, even supportive husbands, family members and friends were not able to fulfil all their emotional needs. Most women in our study did not know about any appropriate peer support groups, but said that they would have appreciated such support.

One woman herself founded a peer support group, which rapidly grew from five to more than 30 through word of mouth and by referrals from fertility clinic counsellors. A second group evolved which included women who meanwhile had become pregnant, and was formed due to the increasing size of the first group and respect for sensitivity of the non-pregnant women. Both support groups were run by the one woman herself struggling with infertility. While this initially fulfilled her own need, with time she had been taking on more responsibilities. Two other women in our study had joined this unique local support group and expressed how the peer group fulfilled a need for a period of time, but that they had to move away to move on themselves.

I feel like Erin Brockovich sometimes with all the information. Everybody’s story is different. (ID 8, primary infertility, aged 39 years, founded peer-led support group)

I thought if I only had known about this group sooner, it probably would have helped me get through a lot of what I’d been going through with the grief and the questioning about IVF. I probably would have survived a bit better. (ID 12, stillbirth, aged 38 years)

The support group was good initially because there are other people who are having this same kind of experience as me and then some of them have babies…and you get left behind with all these new people and their misery and all the ones who have got babies are moving on into this new part of their life. Once the people that I initially met and had really good connections with, got pregnant, for me that was the end. (ID 2, primary infertility, aged 36 years)

When you go there, you just keep hearing the same sad stories after a while. I guess it’s probably a bit like war veterans. You keep going over the same stuff and once you’ve heard it… I didn’t want to tell my story anymore. (ID 2, primary infertility, aged 36 years)

Such peer-led face-to-face support groups did not suit everybody. A couple of women expressed that they preferred to stay private. However, one fifth of our interviewees (n=5) found some emotional support and information in online discussion forums.

### Professional-led support for stillbirth

In contrast to the women experiencing infertility in form of conception problems or early pregnancy loss/recurrent miscarriages (n=22), the three women in our study who experienced late pregnancy loss/stillbirth received professional-led emotional and instrumental support through the hospital and local and national support programs, including ‘SIDS (Sudden Infant Death Syndrome) and Kids’, SANDS (Stillbirth And Neonatal Death Society) and the Teddy Love Club [32-34]. All three women expressed their need and appreciation for such support and highly valued the social recognition of their loss.

In the hospital after the stillbirth, they were fantastic and did so much. Straight away I got the memory box, the gowns, the blankets, the little beanie and the naming certificate and the certificates for his birth, hand and footprints and photos. I was like yeah, bring it on. I want everything.

The hospital gave me a counsellor, she came in and talked and gave me information. I read most of it when I came home, about the funeral, about the virus, about the blood transfusion. (ID 11, stillbirth, aged 33 years)

The SIDS and KIDS counsellor came into our workplace around the first anniversary of losing my first baby and gave a session about the grief process. Our manager allowed this session as part of a bigger meeting, but an optional part, and probably 90% of the people still stayed, men and women, and they said it was useful to understand the grief process. (ID 10, secondary infertility after stillbirth and healthy live birth, aged 36 years)

The Teddy Love Club provides support for miscarriages and stillbirths run by volunteers. You receive a teddy bear named after another child that has passed away and it gives you something to carry out of the hospital rather than leaving with nothing in your arms.

We had recently a walkathon and balloon release in memory of all our babies. I now donate bears in the memory of my little girl. Last year they had a teddy bear run and I put out the call to all my friends and family, even my Facebook friends, and I got over 100 odd bears as a result, so, there’s probably a bear named after my little girl in every hospital now. (ID 12, stillbirth, aged 38 years)

## Discussion

Our study examined women’s experiences of Traditional Chinese Medicine therapy for infertility and viable pregnancy. Women sought TCM on their own accord at any stage of their quest for a child, some in conjunction with IVF. Our observational study explored the motivation and background of women, their quality of life, coping strategies and support needs at the time they sought TCM therapy.

The women in our study reported through questionnaires and interviews compromised quality of life due to the high level of distress, guilt, grief and frustration caused by their infertility, in line with the international literature [28,35,36]. However, our study sample was highly motivated, given that they were actively seeking alternative support for their ‘problem’. They were less accepting of infertility as incurable, while applying similar coping strategies to a comparable group of women who underwent conventional treatment for infertility and who had similar characteristics including age, years of infertility, and infertility history [37].

We recognize that the small study population and self-selection bias limits the generalisability of these findings. All women participating in the study had sought and were satisfied with TCM treatment; those infertile women who did not seek TCM or who may have had negative TCM experiences were not represented. Also, two-thirds of the women had proceeded to TCM after unsuccessful treatment in fertility clinics, and their perceptions of ART procedures may not be representative of successful ART participants. However, our sample size was sufficient to reach thematic saturation, and the findings were consistent with other qualitative studies on related topics [11]. In addition, we validated questionnaire responses with the interviews.

As infertility is not a transient event, ongoing emotional and instrumental support is pivotal to the wellbeing and quality of life of the affected. Women in our study reported being rewarded by TCM therapy to help manage their fertility. TCM therapy increased women’s sense of personal agency and control while reducing powerlessness through continuity of care and education.

The TCM approach to fertility routinely uses characteristics of the menstrual cycle, in addition to general health, pulse and tongue diagnosis to ascertain the underlying pattern of imbalance. The TCM treatment principle is to restore balance, which affects hormonal regulation of the menstrual cycle and provides a physiological environment to facilitate conception, implantation, and maintenance of a viable pregnancy [16,17] (Additional file 2). TCM therapy for women with infertility due to conception problems is based on the same diagnostic principles as women experiencing recurrent miscarriage or stillbirth. With the help of the TCM practitioner, all women in our study learned to understand their fertility status by more careful observation of their menstrual cycles and of the influence of diet and lifestyle. Knowledge of the characteristics of a normal balanced and fertile cycle facilitated treatment compliance and improved emotional wellbeing.

While TCM therapy fulfilled some important support needs not achieved by Western medical approaches to infertility, women in our study also reported a need for support on a larger societal level. Emotional support by their partners, family or friends was not necessarily available to or desired by every woman experiencing infertility or matched the level or quality needed. Nevertheless, many women expressed the need to talk to someone who had a similar experience and understanding, looking for peer support and solicited expert advice. Although the need for these types of support has been reported by others for some time [38-41], a lack of organized social support for infertility remains in Australia.

Most of the women in our study were not aware of an existing peer-led social network support group; however, one of our interviewees founded a local support group out of need, which grew over time, and counsellors at the local fertility clinic made referrals to it.

While online information and discussion forums exist in Australia and were used by women in our study, existing local support groups may be less accessible and relevant for some women. The ACCESS Network in Australia, for example, provides information closely linked to fertility clinics, and contact details to groups limited to specific needs, e.g. single women, donor eggs [5]. Professional support may be limited at some locations, for example, ACCESS lists one fertility support counsellor in South Australia and one in the Australian Capital Territory compared to about 15 in Queensland or Victoria. The ISIS Fertility network links to a few specialised support groups, including Monash IVF, endometriosis, donor sperm groups. The ISIS Fertility network also refers to online support groups run by commercial organisations (for example: http://www.essentialbaby.com.au, http://www.bubhub.com.au, http://www.bellybelly.com.au), but to date does not list local support groups in all states [42].

In contrast, in the US the need for ‘face-to-face’ infertility support groups has been recognized and is supported by a national association. In 2010, the national infertility network ‘RESOLVE’ fostered 122 peer-led support groups in 36 states and districts and 39 professional-led support groups in eight states [43]. Women who would like to start a peer-group are supported with comprehensive material and advice available online and by phone. In addition, RESOLVE engages in raising fertility awareness on multiple levels, including promotion of events such as the ‘National Infertility Awareness Week’, ‘Walk of Hope – Help breaking the silence’, ‘Advocacy Day – Stop being ignored’, charity events and recognition awards ‘Night of Hope’, and family-building conferences. In 2010, for example, three conferences were held with 375 attendees [43].

Other researchers reported that professional-led support groups consisting of 2–6 monthly two-hour structured sessions of art therapy helped with reducing isolation, decision making of treatment steps, and better coping and self-acceptance of infertile couples [44,45].

The support groups in Australia available to help parents who have had a stillbirth with their grieving process, break their isolation, and help to connect to others in similar situations [32-34]. In addition, these national and local programs raise awareness about the problem and facilitate an open dialogue stimulating communication, understanding and support also from those not directly affected, including family, friends, colleagues and the wider community.

In Australia, infertility does not have the same depth of organizational support as stillbirth has. Our research indicated, however, that greater fertility awareness and understanding of the infertility problem is needed. The infertility network in the US, RESOLVE, could be a model for raising (in-) fertility awareness and providing support for those in need, and could facilitate dissemination of evidence of approaches to fertility management, including Traditional Chinese Medicine.

## Conclusions

Infertility decreases quality of life for some women and requires ongoing emotional and instrumental support for them. Traditional Chinese Medicine increased women’s sense of personal agency and control through continuity of care and education not routinely available in the Western model of care. Peer-led and professional-led support groups governed by a national network are also needed for women and couples experiencing infertility to help break isolation and raise awareness of integrative approaches to fertility management.

## Abbreviations

ART: Artificial reproductive technology; IVF: In vitro fertilisation; TCM: Traditional Chinese medicine; TLMK: Tuebingen quality of life questionnaire.

## Competing interests

The authors declare that they have no competing interests.

## Authors’ contributions

KR conceptualised the study, secured funding, and recruited study participants. AA conducted the interviews. Both authors undertook data analysis and interpretation. KR prepared the manuscript with contributions from AA. All authors approved the final version.

## Pre-publication history

The pre-publication history for this paper can be accessed here:

http://www.biomedcentral.com/1472-6874/13/17/prepub

## Supplementary Material

Additional file 1 Quality of life questionnaire. Click here for file

Additional file 2 Checklist of basic fertility indicators. Click here for file
