# Supplementary material for: Quality of life, coping strategies and support needs of women seeking Traditional Chinese Medicine for infertility and viable pregnancy in Australia: a mixed methods approach
Source: BMC Womens Health. 2013 Apr 9;13:17. doi: 10.1186/1472-6874-13-17 (PMC3635943; doi:10.1186/1472-6874-13-17)
Supplement: Additional file 1 — Quality of life questionnaire. [file 1472-6874-13-17-S1.doc]

**Appendix 1: Quality of life questionnaire (Gebert 2004; Schanz 2005)**

Respond to each of the following items by circling the number on the scale from 1 = ‘strongly disagree’ to 5 = ‘strongly agree’:

|  | **Questions** | **I strongly disagree** | **I disagree** | **I neither agree or disagree** | **I agree** | **I strongly agree** |
| --- | --- | --- | --- | --- | --- | --- |
| 1. | Not having been able to mother a child is distressing to me. | **1** | **2** | **3** | **4** | **5** |
| 2. | I can’t achieve happiness without a child. | **1** | **2** | **3** | **4** | **5** |
| 3. | I feel hurt when others make remarks about our childlessness. | **1** | **2** | **3** | **4** | **5** |
| 4. | I feel upset when I see a baby pusher / stroller. | **1** | **2** | **3** | **4** | **5** |
| 5. | I consider infertility a personal shortcoming. | **1** | **2** | **3** | **4** | **5** |
| 6. | I feel down. | **1** | **2** | **3** | **4** | **5** |
| 7. | I am coping well with our difficulties conceiving. | **1** | **2** | **3** | **4** | **5** |
| 8. | My life revolves around trying to have children. | **1** | **2** | **3** | **4** | **5** |
| 9. | Planning of our future has been hindered by our difficulties conceiving. | **1** | **2** | **3** | **4** | **5** |
| 10. | I avoid contact with people who have children. | **1** | **2** | **3** | **4** | **5** |
|  |  |  |  |  |  |  |
| 11. | The psychological stress interferes with work, leisure activities. | **1** | **2** | **3** | **4** | **5** |
| 12. | I feel like giving up. | **1** | **2** | **3** | **4** | **5** |
| 13. | Certain aspects of my relationship with my partner have become less important since we have had problems conceiving. | **1** | **2** | **3** | **4** | **5** |
| 14. | My partner and I have less sex when I am not ovulating. | **1** | **2** | **3** | **4** | **5** |
| 15. | The stress of trying to conceive decreases feelings of tenderness for my partner. | **1** | **2** | **3** | **4** | **5** |
| 16. | The desire to have a child decreases my sexual desire for my partner. | **1** | **2** | **3** | **4** | **5** |
| 17. | Sex is a chore. | **1** | **2** | **3** | **4** | **5** |
| 18. | I feel under pressure when I am ovulating. | **1** | **2** | **3** | **4** | **5** |
| 19. | I feel less satisfied after sex than I used to before we were trying to conceive. | **1** | **2** | **3** | **4** | **5** |
| 20. | I feel like a failure because of our problems conceiving. | **1** | **2** | **3** | **4** | **5** |
|  |  |  |  |  |  |  |
| 21. | I feel guilty for having let my partner down. | **1** | **2** | **3** | **4** | **5** |
| 22. | I have feelings of low self worth. | **1** | **2** | **3** | **4** | **5** |
| 23. | I can better my life situation. | **1** | **2** | **3** | **4** | **5** |
| 24. | I can enjoy the good things in life. | **1** | **2** | **3** | **4** | **5** |
| 25. | I have a good sense of humour about life. | **1** | **2** | **3** | **4** | **5** |
| 26. | I have lost interest in things I used to enjoy. | **1** | **2** | **3** | **4** | **5** |
| 27. | I am able to relax. | **1** | **2** | **3** | **4** | **5** |
| 28. | I am satisfied with my life. | **1** | **2** | **3** | **4** | **5** |
| 29. | I have difficulties with planning and problem solving. | **1** | **2** | **3** | **4** | **5** |
