# Supplementary material for: Quality of life, coping strategies and support needs of women seeking Traditional Chinese Medicine for infertility and viable pregnancy in Australia: a mixed methods approach
Source: BMC Womens Health. 2013 Apr 9;13:17. doi: 10.1186/1472-6874-13-17 (PMC3635943; doi:10.1186/1472-6874-13-17)
Supplement: Additional file 2 — Checklist of basic fertility indicators. [file 1472-6874-13-17-S2.doc]

**Appendix 2: Checklist of basic fertility indicators**

**A fertile/balanced menstrual cycle is ideally:**

- between 28 and 30 days long
- with menstruation lasting 5 days
- the blood is
  - neither too runny nor too sticky
  - deep red in colour, almost as if caused by a cut
  - without clots
  - without pain or spotting experienced at any part of the cycle
- the basal body temperature (BBT) should not waver for more than 0.1 degrees Celsius in each phase
  - with the BBT in the follicular phase hovering around 36.2-36.5°
  - rising up to 36.7-37.0° in the luteal phase
- ovulation should occur on day 12 or 13
  - with a thermal shift of 0.3-0.5° in 1-2 days
- cervical mucus is observed throughout the cycle
  - with runny, cloudy mucus during infertile days
  - with stretchy, clear mucus during fertile days around ovulation

**Examples for infertile/imbalanced menstrual cycles are:**

- dark purple coloured clotted menstrual blood linked with heavy flow and long cycles
- pink watery scanty flow of blood
- a slow rise of temperature/extended thermal shift of more than 2 days from follicular to luteal phase
- absence of fertile mucus

**Table legend:** Observable imbalances of the menstrual cycle are associated with TCM pattern diagnosis, such as Liver Qi Stagnation, Spleen Deficiency or Damp-Heat, and can be addressed with TCM herbal therapy, acupuncture and lifestyle changes.
